# Phrenic Nerve Injury After Catheter Ablation of Atrial Fibrillation

**Published:** 2007-01-01

**Authors:** Frederic Sacher, Pierre Jais, Kent Stephenson, Mark D O'Neill, Meleze Hocini, Jacques Clementy, William G Stevenson, Michel Haissaguerre

**Affiliations:** 1 CHU de Bordeaux/ Universite Bordeaux II, France; 2 Brigham and Women's Hospital/ Harvard Medical School, Boston, USA

**Keywords:** Phrenic nerve injury, Catheter ablation, Atrial Fibrillation

## Abstract

Phrenic Nerve Injury (PNI) has been well studied by cardiac surgeons. More recently it has been recognized as a potential complication of catheter ablation with a prevalence of 0.11 to 0.48 % after atrial fibrillation (AF) ablation. This review will focus on PNI after AF ablation

Anatomical studies have shown a close relationship between the right phrenic nerve and it's proximity to the superior vena cava (SVC), and the antero-inferior part of the right superior pulmonary vein (RSPV). In addition, the proximity of the left phrenic nerve to the left atrial appendage has been well established.

Independent of the type of ablation catheter (4mm, 8 mm, irrigated tip, balloon) or energy source used (radiofrequency (RF), ultrasound, cryothermia, and laser); the risk of PNI exists during ablation at the critical areas listed above.

Although up to thirty-one percent of patients with PNI after AF ablation remain asymptomatic, dyspnea remain the cardinal symptom and is present in all symptomatic patients. Despite the theoretical risk for significant adverse effect on functional status and quality of life, short-term outcomes from published studies appear favorable with 81% of patients with PNI having a complete recovery after 7 ± 7 months.

**Conclusion:**

Existing studies have described PNI as an uncommon but avoidable complication in patients undergoing pulmonary vein isolation for AF. Prior to ablation at the SVC, antero-inferior RSPV ostium or the left atrial appendage, pacing should be performed before energy delivery. If phrenic nerve capture is documented, energy delivery should be avoided at this site. Electrophysiologist's vigilance as well as pacing prior to ablation at high risk sites in close proximity to the phrenic nerve are the currently available tools to avoid the complication of PNI.

## Introduction

Phrenic Nerve Injury (PNI) has been well studied by cardiac surgeons [1-3]. Protective measures during cardiac surgery has led to significant decline in the incidence of PNI from 30%-55% [2] during the early 1980s to 10% in the last few years [3].

Recently the association of PNI after percutaneous based catheter ablation procedures such as: left lateral Wolff Parkinson White [4], inappropriate sinus tachycardia [5] and atrial fibrillation (AF) [6-8] has sparked renewed interest. Currently the reported prevalence of PNI as a complication of AF ablation is estimated between 0.11% to 0.48% [7,9]. This review will focus on PNI after AF ablation, we will revisit some fundamental anatomical concepts, and technical considerations associated with PNI. In addition, we will summarize presenting symptoms, outcomes and proposed strategies to avoid this complication.

## Anatomy

In terms of proximity to cardiac structures relevant to electrophysiologists, the right phrenic nerve is close to the superior vena cava (SVC) superiorly and adjacent to the lateral border of the entrance of the inferior vena cava (IVC) to the right atrium inferiorly. While the right phrenic nerve is immediately adjacent to the anterolateral wall of the SVC, it veers posteriorly as it approaches the superior cavoatrial junction. More inferiorly, it passes close to the junction of the left atrium to the right superior pulmonary vein (RSPV). Sanchez-Quintana et al. [10] found that the anterior wall of the RSPV is <2 mm from the right phrenic nerve in 32% of their autopsy series.

**Figure 1:**
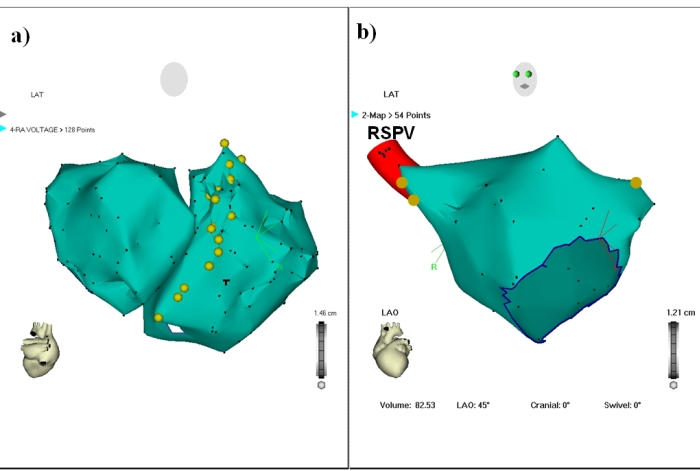
Endocardial site where phrenic nerve was captured in patients with phrenic nerve injury who had a redo procedure and completely recovered. (A) Endocardial right phrenic nerve course in the right atrium (RA) (posteroanterior view on anatomic Carto map). (B) Site where right and left phrenic nerve were captured in the left atrium (left anterior oblique [LAO] view on anatomic Carto map). RSPV: right superior pulmonary vein. Figure 3 from Journal of American College of Cardiology, V47 (12): 2502, Sacher F et al: "Phrenic nerve injury after AF ablation…" © 2006 The American College of Cardiology Foundation.

Figure 1 shows an example of sites with phrenic nerve capture in a woman with phrenic nerve injury during AF ablation. As demonstrated by Sanchez-Quintana et al. [10], there is close vicinity between right phrenic nerve and SVC and anterior part of the RSPV.

Whereas much less common (2/32(6%)- cf Table 1), left PNI could also occur during RF delivery at the proximal left atrial appendage roof [7]. In terms of anatomy, the left phrenic nerve lies over the left atrial appendage. Its course then runs along the pericardium overlying the left ventricle (LV). In front of the root of the left lung, the left phrenic nerve is located between the fibrous pericardium that covers the anterolateral face of the LV and the mediastinal pleura, and it takes an oblique passage downward to reach the diaphragm behind the tip of the LV. The relationships of the nerve to the left heart structures depended on whether it descends along a path related to the anterior surface of the LV or passes leftward along a course related to the obtuse margin of the LV.

## Technical considerations

The use of a non-RF source of energy is unlikely to prevent this complication since PNI have been reported with RF but also with ultrasound [7,8,11], laser [8,12], cryotherapy [8,13]. Heat as well as cold may have a deleterious effect on phrenic nerve. Ice slush during cardioplegia was recognized for many years by cardiac surgeons as a risk factor for PNI [2]. Sarabanda et al. [14] found right PNI in 50% of their dogs undergoing cryothermal isolation of the pulmonary vein using a balloon technique.

Heat energy can also have adverse effects, Bunch et al. [15] showed a correlation between nerve injury and the temperature achieved during RF delivery. One striking point was the discrepancy between temperature achieved at the ablation site (RSPV) and the markedly higher temperatures achieved at the phrenic nerve in one dog with PNI. Accurate tissue temperature monitoring is one of the major problems of catheter ablation. Currently, only the catheter tip temperature is monitored, which can be vastly different from the core tissue temperature [15]. A reliable tool to monitor tissue temperature during energy delivery theoretically could improve efficacy and safety of such procedure. However, immediate nerve injury could occur thus suggesting putative mechanisms other than solely direct heat or cold transfer. It has been proposed that nerve dysfunction could be related directly to electrical current (electromagnetic field generated at the catheter tip [16], generation of a resonance current around the heart [17]).

This complication has been observed with a variety of catheters (standard 4 mm, 8 mm, 4mm irrigated tip and balloon) however it seems that the balloon design is more prone to cause PNI (in 6 to 50% of the procedure [11] [14]).

Another interesting point is that PNI occurs independent of the strategy of AF ablation used (pulmonary vein isolation vs. wide anatomical circumferential ablation) [7].  This is not so surprising since the lesions are made in the same anatomic areas, the only essential difference between both methods is the resultant end-point and whether or not electrical PV disconnection occurs.

## Symptoms

There is a large spectrum of symptoms depending on the patient and an underlying disease (chronic obstructive pulmonary disease). Patients can be asymptomatic in 10/32 (31%), however the most frequent symptoms is dyspnea or shortness of breath 22/32 [6-8] which is present in all of the symptomatic patients. Others symptoms or clinical findings are cough or hiccup during ablation and the development of post-ablation pneumonia or pleural effusion. In asymptomatic patients, the diagnosis is made on the routine chest X-ray with hemi diaphragm paresis or paralysis (hemidiaphragm elevation with paradoxical movement).

## Outcomes

Published outcomes appear favorable. In one study by Bai et al. [8], all of their 13 patients had completely recovered. In our study, [7] 3 (17%) patients had partial recovery and then become asymptomatic but 3 (17%) patients failed to recover.  Of the patients who had persistent PNI, two patients had no AF recurrence and are symptomatically better than before ablation but one had persistent limiting dyspnea and underwent plication of the right diaphragm 18 months after the AF ablation. This procedure resulted in some symptomatic improvement associated with restored right lung volume but persistent diaphragmatic paralysis.

Recovery time is widely variable, ranging from 1 day to 28 months (mean: 7 ± 7 months). Except for early interruption of energy delivery no other parameter was predictive of early recovery. However phrenic nerve function can still improve more than 1 year after the procedure.

In our experience, pulmonary rehabilitation seems interesting and may have some role in favorable recovery especially in patients with prior lung disease.

## How to avoid this complication?

Ablation of specific structures appear to be more likely associated with PNI and warrants greater caution during ablation (Figure 1); these include the infero-anterior part of right PV ostium, the postero-septal part of the SVC, and the proximal left atrial appendage roof. In these areas high output pacing should be performed before RF delivery. In case of diaphragmatic stimulation, energy application at this site should be avoided. Early suspicion of PNI should be considered in the case of hiccup, cough or decrease in diaphragmatic excursion during  energy delivery. Early recognition of PNI during RF delivery allows the immediate interruption of the application, which is associated with the rapid recovery of phrenic nerve function.

## Conclusion

PNI although an infrequent complication associated with AF ablation (0.11 to 0.48%) has been observed with the use of a variety of catheters (4-mm, 8-mm, irrigated tip, balloon) and energies (RF, cryo, ultrasound, laser) suggesting that only physician "vigilance" may minimize the risk of irreversible PNI. The critical areas are the right PV (antero-inferior), the SVC, and the left atrial appendage. Complete or partial recovery of diaphragmatic function was observed in most patients.

## Figures and Tables

**Table 1:**
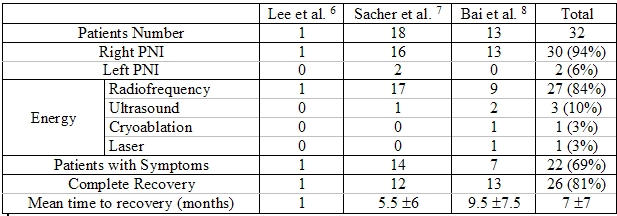
Characteristics of Phrenic Nerve Injury in the different studies
